# Anti-*N*-methyl-d-aspartate receptor encephalitis induced by bilateral ovarian teratomas with distinct histopathologic types: A case report and brief literature review

**DOI:** 10.1097/MD.0000000000018148

**Published:** 2019-11-27

**Authors:** Wenchen Li, Dan Jia, Lan Tong, Zhijun Lun, Hailiang Li

**Affiliations:** a Department of Neurotrauma; b Department of Urology; c Department of Radiology; d Department of Library; e Department of Outpatient, The First Hospital of Jilin University, Changchun, Jilin, China.

**Keywords:** anti-*N*-methyl-d-aspartate receptor encephalitis, bilateral teratomas, immature teratoma, mature teratoma, ovarian teratoma

## Abstract

**Rationale:**

Anti-*N*-methyl-d-aspartate receptor (NMDAR) encephalitis is an autoimmune disorder that is most frequently induced by ovarian teratoma in young females. The condition can be controlled and reversed via ovarian tumor resection and immunotherapy. However, anti-NMDAR encephalitis induced by bilateral ovarian teratomas with distinct histopathologic types is rarely reported in the literature.

**Patient concerns:**

A 23-year-old woman presented with seizures.

**Diagnoses:**

The diagnosis was anti-NMDAR encephalitis associated with ovarian teratomas based on positive anti-NMDAR antibody tests in both the cerebrospinal fluid and serum, and the detection of bilateral ovarian lesions on pelvic computed tomography. The postoperative histopathologic examination confirmed that the left lesion was an immature teratoma, and the right lesion was a mature teratoma.

**Interventions:**

We performed surgical resection of the ovarian teratomas and administered immunotherapy for the control of anti-NMDAR encephalitis. Chemotherapy was administered for the immature teratoma.

**Outcomes:**

The patient recovered without any postoperative complications. She has been confirmed to be in complete clinical remission, and has not had a recurrence during 18 months of follow-up.

**Lessons:**

Anti-NMDAR encephalitis induced by bilateral ovarian teratomas of differing histopathologic types (1 immature and 1 mature) is rare. Early diagnosis and treatment with tumor resection, immunotherapy, and chemotherapy are critical for a good prognosis.

## 1 Introduction

Anti-*N*-methyl-d-aspartate receptor (NMDAR) encephalitis is a severe and potentially fatal condition. It was 1st identified by Dalmau and Bataller in 2007 as a synaptic autoimmune disease in which anti-NMDAR antibodies are detectable in the serum or cerebrospinal fluid (CSF).^[1]^ In 2011, a study of 501 patients with anti-NMDAR encephalitis found that 38% of patients had concomitant tumors.^[2]^ A recent review of 432 cases of anti-NMDAR encephalitis published in the PubMed database until March 2017 found that of the 293 female patients, 68 (23%) were diagnosed with ovarian teratoma.^[3]^ In 2014, a systematic study^[4]^ evaluated 134 patients with ovarian teratoma-associated anti-NMDAR encephalitis and a known histopathologic tumor type. It found that 74% of the patients had mature teratomas (dermoid cysts), and 21.6% had immature teratomas; moreover, only 3 of the 15 patients with bilateral ovarian teratomas had both a mature and an immature teratoma.

Herein, we present a rare case of anti-NMDAR encephalitis induced by bilateral ovarian teratomas, 1 mature and 1 immature. We also review the literature on anti-NMDAR encephalitis induced by bilateral ovarian teratomas.

## 2 Case report

**Figure 1:**
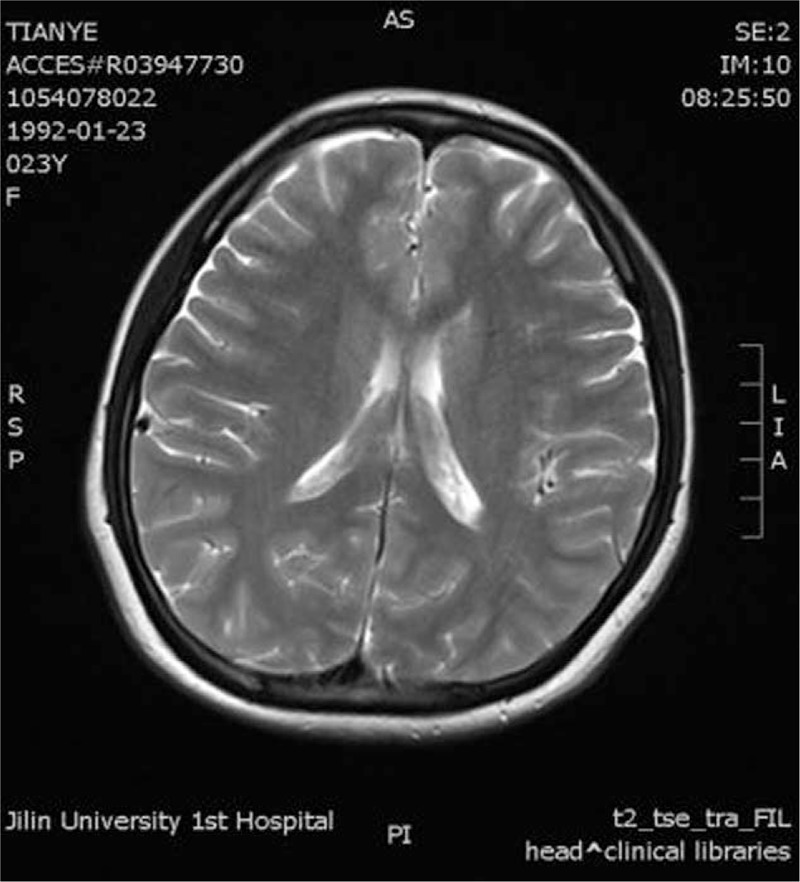
Craniocerebral magnetic resonance imaging showing no abnormality.

This study was approved by the ethics committee of the First Hospital of Jilin University, and the written informed consent was obtained from the patient. A 23-year-old woman was admitted to the emergency department of our hospital with a main complaint of seizures. She had 1st experienced a seizure 5 days before admission. The 1st seizure had lasted for 10 minutes and had been accompanied with a total loss of consciousness, binocular superduction, foaming at the mouth, odaxesmus, flexion of the upper limbs, stretch jitter of the lower limbs, and urinary incontinence. At the time, she was immediately brought to the radiology department of our hospital for craniocerebral magnetic resonance imaging (MRI), but no abnormality was detected (Fig. 1), and no special treatment was employed. Three days before admission, she again developed the same symptoms, and 1 day before admission, she developed disordered speech. She was immediately brought to the emergency department and then transferred to the neurologic department of our hospital with a primary diagnosis of “suspected encephalitis.” She had a history of upper respiratory tract infection that began 2 days before admission.

**Figure 2:**
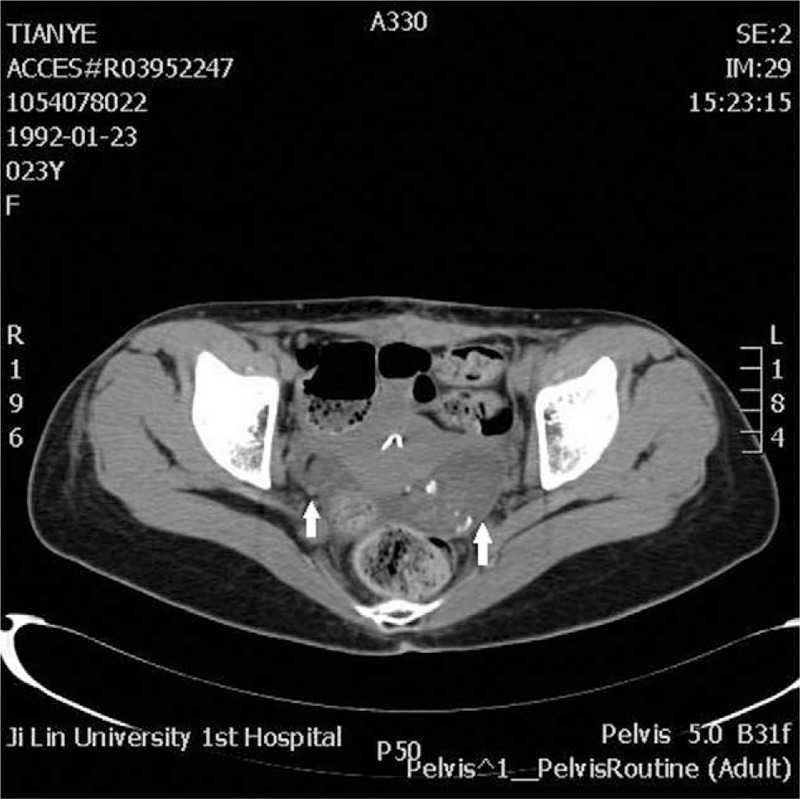
Bilateral ovarian lesions (arrows). Left tumor, 5.4 × 3.2 cm; right tumor, 3.1 × 1.8 cm.

On admission, a physical examination revealed the following: blood pressure, 118/68 mm Hg; heart rate, 96/min; coma state with poor compliance to examination; round pupils of equal size (*d* ≈ 3.0 mm) with preserved direct/indirect light reflexes; movable limbs with grade 3 muscle strength and normal muscle tension; bilateral positive Babinski sign; cervical rigidity (2 fingers); and positive meningismus. Other physical examination results were unavailable due to poor compliance. CSF examination showed the following: fungal smear, no hyphae/spores; tubercle bacilli smear, acid-fast bacilli (−); special bacterial smear (India ink stain), *Cryptococcus neoformans* (−); general bacterial examination: bacteria (−); chlorine, 133.5 mmol/L; and white blood cells: 71 × 10^6^/L. Immunohistochemical tests for autoimmune antibodies were performed using electrochemiluminescence immunoassay (UniCel DX1800; Beckman Coulter, Brea, California). The results were as follows: CSF anti-NMDAR antibody (+) and serum anti-NMDAR antibody (+). Electroencephalography (EEG) showed mainly slow waves. The diagnosis was revised to anti-NMDAR encephalitis, and pelvic computed tomography (CT) was performed to look for ovarian teratomas. The CT scans revealed bilateral ovarian lesions, with the left lesion measuring 5.4 × 3.2 cm, and the right one measuring 3.1 × 1.8 cm (Fig. 2).

**Figure 3:**
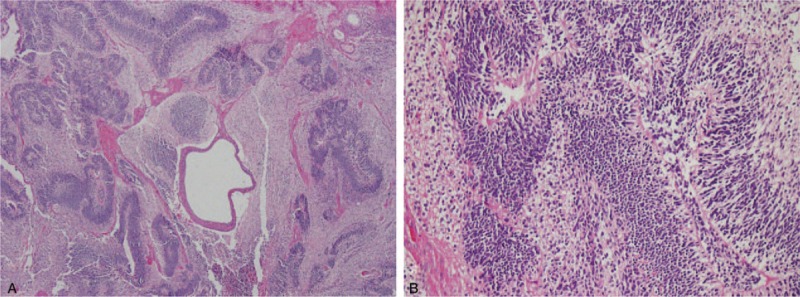
Pathologic examination of left ovarian teratoma. (A) Magnification, 4×. (B) Magnification, 40×.

A laparotomy was performed for the resection of the ovarian lesions. During the surgery, the ovarian lesions appeared smooth and were of distinct sizes, with the left one being about the size of 5.0 × 4.0 × 4.0 cm and the right one about the size of 3.0 × 3.0 × 2.5 cm. Both lesions were completely resected and subjected to intraoperative frozen-section examination, which showed an immature teratoma (left, no less than grade II) and a mature teratoma (right). Because of the malignant nature of the immature teratoma, we performed the following staging operations: extended excision including left adnexectomy, pelvic lymphadenectomy, para-aortic lymphadenectomy, and omentectomy. Bilateral salpingo-oophorectomy was not performed to preserve fertility. Written informed consent for the above surgical measures had been obtained prior to the operation. After the operation, the patient was transferred to an intensive care unit for comprehensive treatment, which included infection prevention, circulation improvement, neural nutrition, sedation, and 1st-line immunotherapy. The preoperative sporadic convulsive seizures and the other accompanying symptoms were gradually and successfully controlled. A postoperative pathologic examination revealed the following: left ovarian immature solid-cystic teratoma (grade II/III); right ovarian mature cystic teratoma; and no tumor metastasis in tissue samples from the left fallopian tube, omentum, peritoneum, and lymph nodes. The histopathologic appearance of the left ovarian immature teratoma was in accordance with common diagnostic standards, since a mass of primitive neuroglial elements was found to be mingled within surrounding lymphocytes and ectodermal elements, including squamous epithelium, sebaceous glands, and mesodermal ingredients (Fig. 3).

The patient was rehabilitated and transferred to the general ward to undergo comprehensive treatment with 1st-line chemotherapy. She was discharged on the 36th day after the operation. No 2nd-line chemotherapy or postoperative cerebrocranial structural/resting-state MRI was implemented due to the patient's personal written refusal. The patient was confirmed to be in complete clinical remission and has not had a recurrence during 18 months of follow-up. Informed written consent was obtained from the patient for publication of this case report and accompanying images.

## 3 Discussion

Anti-NMDAR encephalitis is an autoimmune disorder that is most frequently induced by ovarian teratoma in young girls/women.^[4]^ Most patients with anti-NMDAR encephalitis induced by ovarian teratomas are diagnosed with unilateral mature ovarian teratomas.^[5,6]^ Anti-NMDAR encephalitis caused by bilateral ovarian teratomas is rare. According to a systematic review^[4]^ of all cases of ovarian teratoma-associated anti-NMDAR encephalitis published in the PubMed and SCOPUS databases until 2014, with no language restrictions, only 20 cases were caused by bilateral tumors. In 5 of these 20 cases, the histopathologic type was not specified; in the remaining 15 cases, the histopathologic type was mature teratoma (10 cases), immature teratoma (2 cases), and 1 mature and 1 immature teratoma (3 cases). We searched the PubMed and CNKI databases for all cases of anti-NMDAR encephalitis induced by bilateral ovarian teratomas in which the histopathologic type was specified. We limited our search to articles published in English or Chinese between December 1, 2013 and December 31, 2018. We found 8 such cases,^[7–13]^ which have been summarized in Table 1.

**Table 1:**
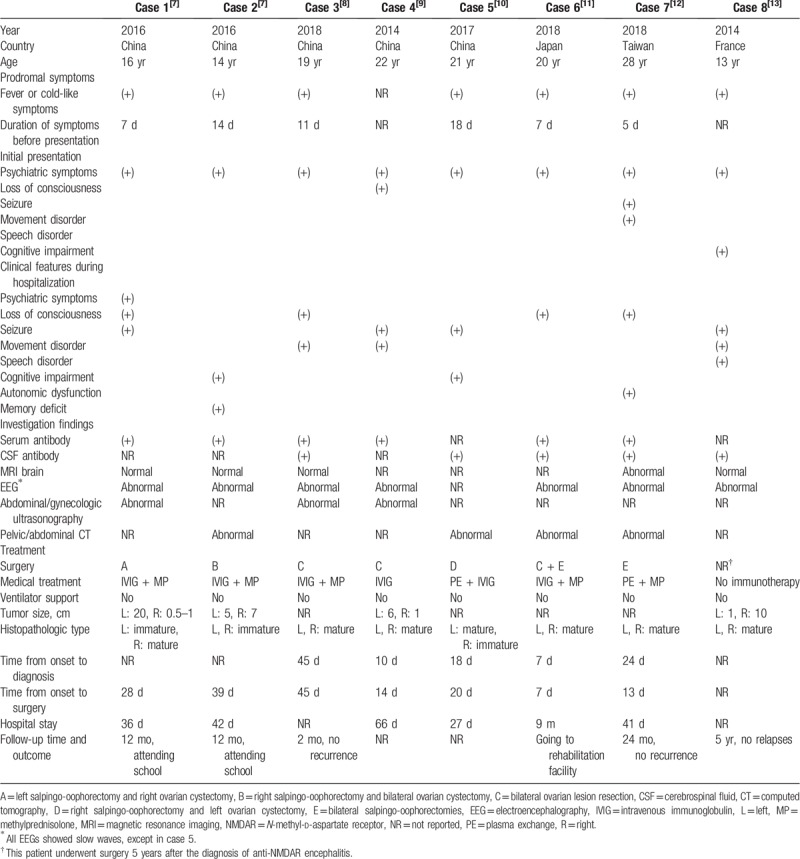
Demographic, clinical, investigation, and treatment details of patients with anti-NMDAR encephalitis induced by bilateral ovarian teratomas.

Among these 8 cases and the present case, there were 5 cases of mature teratomas and 4 cases of 1 mature and 1 immature teratoma. Therefore, among the 24 patients (15 patients in the previous review and 9 patients in the present review) with anti-NMDAR encephalitis induced by bilateral ovarian teratomas, 15 patients had mature teratomas, 2 patients had immature teratomas, and 7 patients had 1 mature and 1 immature teratoma. In our patient, primitive neuroglial elements were detected in the left immature ovarian teratoma and not in the right mature ovarian teratoma, although mature ovarian teratomas usually contain neuroglial elements. Regardless of the histologic type, teratomas that contain neural tissue could trigger an immune response resulting in the overproduction of anti-NMDAR antibodies. These antibodies react with NMDARs mainly in the hippocampus and forebrain regions, and trigger a cascade of symptoms recognized as anti-NMDAR encephalitis.^[6]^ The pathologic mechanism underlying bilateral ovarian teratoma-associated anti-NMDAR encephalitis may involve the neuroglial element of the tumors, especially in patients with an immature and a mature ovarian teratoma, and this should be explored in future research.

The clinical manifestations of anti-NMDAR encephalitis can be divided into 4 specific phases: prodromal phase, psychotic phase, neurologic-complications phase, and recovery phase.^[12,14,15]^ Of the 9 patients in our review, 8 had prodromal symptoms such as fever. Patients are unlikely to be diagnosed at this stage, as prodromal symptoms are nonspecific and mimic the symptoms of common cold. Behavioral change is the most frequent manifestation in the psychotic phase, and patients are often brought to the psychiatry department in this stage. In our review, 8 of the 9 patients had psychotic symptoms, though our patient had no obvious psychotic symptoms. The neurologic complications of anti-NMDAR encephalitis include unresponsiveness, hyperkinesia, and seizures; patients are often admitted to the neurologic department in this phase. In our review, 3 patients, including the present patient, were admitted to a hospital because of seizures. One study has reported that head injury may be responsible for lowering the threshold for neurologic deficits caused by bilateral ovarian teratoma-associated anti-NMDAR encephalitis.^[12]^ Therefore, accurate diagnosis is difficult and time-consuming, and often neurologic deterioration, and even death, can occur before a diagnosis is reached. The recovery phase can be induced by immunotherapy and tumor resection.

In patients with anti-NMDAR encephalitis, MRI often does not show any abnormality. When abnormal findings are present, they most commonly include medial temporal and frontal hyperintensities on T2/FLAIR imaging, and leptomeningeal contrast enhancement.^[16]^ Accordingly, impaired hippocampal functional connectivity, decoupling of the medial temporal default-mode networks, and overall impairment of the frontotemporal connections can be detected by structural and resting-state functional MRI.^[17]^ The EEG findings are often nonspecific, with slow waves. Of the 9 patients in our review, 8 patients had undergone EEG, which showed abnormal slow waves in all 8 patients. For the diagnosis of anti-NMDAR encephalitis, it is crucial that NMDAR antibodies be detected in the serum or CSF. In female patients, abdominopelvic CT and transvaginal ultrasonography can be performed to identify small ovarian teratomas and confirm the diagnosis. Heightened awareness of this condition, particularly among gynecologists and neurologists, is required to enable the rapid identification of ovarian teratoma-associated anti-NMDAR encephalitis.

The treatments of encephalitis induced by ovarian teratomas include tumor resection, immunotherapy, and symptomatic treatment.^[3,4]^ The most frequent surgical procedure for mature teratoma removal was simple tumor excision while uni/bilateral salpingo-oophorectomy and radical surgery was reserved for patients with immature teratomas.^[4]^ In 4 cases of bilateral mature teratomas in Table 1, 2 cases were performed for simple bilateral ovarian lesion resection and 2 cases was performed for bilateral salpingo-oophorectomies. Two cases of 1 mature and 1 immature teratomas in Table 1 were performed for ovarian lesion resection for mature teratoma and salpingo-oophorectomy for immature teratoma. For our patient, we performed tumor removal for the right mature ovarian teratoma and radical resection for the left immature ovarian teratoma. First-line immunotherapy consists of corticosteroids, immunoglobulins, plasma exchange, and plasmapheresis. Second-line immunotherapy includes rituximab, cyclophosphamide, mycophenolate mofetil, azathioprine, and methotrexate.^[3,12]^ In addition, chemotherapy is applied in some patients with immature teratoma to prevent recurrence of the tumor. Our patient rapidly improved after surgery combined with 1st-line immunotherapy and chemotherapy, which confirmed that the anti-NMDAR encephalitis had been induced by the bilateral ovarian teratomas.

A recent systematic review reported that in 68 patients with anti-NMDAR encephalitis induced by ovarian teratomas, 65 patients (95.6%) survived while 3 patients (4.4%) died.^[3]^ Acien et al^[4]^ reported that in 174 patients with anti-NMDAR encephalitis induced by ovarian teratomas, 142 (81.6%) cases had full recovery, 20 (11.5%) cases had partial recovery, and 12 (6.9%) cases died. Additionally, in 6 patients with mature and immature teratomas, 4 cases full recovery, 1 case partial recovery, and 1 case died.^[4]^ In Table 1, 6 in 8 cases had recovery and no recurrence during follow-up time. In our patient, she had a good recovery with no relapses during 18 months of follow-up via total resection of the bilateral ovarian teratomas in the early stage and immunotherapy. Early accurate diagnosis and prompt therapy are crucial for a good prognosis in patients.

## Acknowledgment

The authors thank the patient, her family, and Shan Zong, PhD, for providing their information for this study.

## Author contributions

**Data curation:** Dan Jia, Lan Tong, Hailiang Li.

**Supervision:** Wenchen Li, Zhijun Lun.

**Validation:** Dan Jia, Hailiang Li.

**Writing – original draft:** Wenchen Li.

**Writing – review & editing:** Dan Jia, Lan Tong, Zhijun Lun, Hailiang Li.
